# Medical practitioner perspectives on AI in emergency triage

**DOI:** 10.3389/fdgth.2023.1297073

**Published:** 2023-12-06

**Authors:** Beverley A. Townsend, Katherine L. Plant, Victoria J. Hodge, Ol’Tunde Ashaolu, Radu Calinescu

**Affiliations:** ^1^ York Law School, University of York, York, United Kingdom; ^2^ Faculty of Engineering & Physical Sciences, University of Southampton, Southampton, Hampshire, United Kingdom; ^3^ Department of Computer Science, University of York, York, United Kingdom; ^4^ York and Scarborough Teaching Hospitals, York, United Kingdom

**Keywords:** Diagnostic AI System for Robot-Assisted A & E Triage (DAISY), emergency department triage, perceptions, attitudes, medical practitioners

## Abstract

**Introduction:**

A proposed Diagnostic AI System for Robot-Assisted Triage (“DAISY”) is under development to support Emergency Department (“ED”) triage following increasing reports of overcrowding and shortage of staff in ED care experienced within National Health Service, England (“NHS”) but also globally. DAISY aims to reduce ED patient wait times and medical practitioner overload. The objective of this study was to explore NHS health practitioners' perspectives and attitudes towards the future use of AI-supported technologies in ED triage.

**Methods:**

Between July and August 2022 a qualitative-exploratory research study was conducted to collect and capture the perceptions and attitudes of nine NHS healthcare practitioners to better understand the challenges and benefits of a DAISY deployment. The study was based on a thematic analysis of semi-structured interviews. The study involved qualitative data analysis of the interviewees' responses. Audio-recordings were transcribed verbatim, and notes included into data documents. The transcripts were coded line-by-line, and data were organised into themes and sub-themes. Both inductive and deductive approaches to thematic analysis were used to analyse such data.

**Results:**

Based on a qualitative analysis of coded interviews with the practitioners, responses were categorised into broad main thematic-types, namely: trust; current practice; social, legal, ethical, and cultural concerns; and empathetic practice. Sub-themes were identified for each main theme. Further quantitative analyses explored the vocabulary and sentiments of the participants when talking generally about NHS ED practices compared to discussing DAISY. Limitations include a small sample size and the requirement that research participants imagine a prototype AI-supported system still under development. The expectation is that such a system would work alongside the practitioner. Findings can be generalisable to other healthcare AI-supported systems and to other domains.

**Discussion:**

This study highlights the benefits and challenges for an AI-supported triage healthcare solution. The study shows that most NHS ED practitioners interviewed were positive about such adoption. Benefits cited were a reduction in patient wait times in the ED, assistance in the streamlining of the triage process, support in calling for appropriate diagnostics and for further patient examination, and identification of those very unwell and requiring more immediate and urgent attention. Words used to describe the system were that DAISY is a “good idea”, “help”, helpful, “easier”, “value”, and “accurate”. Our study demonstrates that trust in the system is a significant driver of use and a potential barrier to adoption. Participants emphasised social, legal, ethical, and cultural considerations and barriers to DAISY adoption and the importance of empathy and non-verbal cues in patient interactions. Findings demonstrate how DAISY might support and augment human medical performance in ED care, and provide an understanding of attitudinal barriers and considerations for the development and implementation of future triage AI-supported systems.

## 1. Introduction

Emergency Department (“ED”) overcrowding is a major global healthcare concern whose negative consequences are described as both a patient safety issue and a worldwide public health problem (1–3). ED overcrowding results from increased volumes of patients waiting to be seen, delays in assessing or treating patients already presenting in the ED, and barriers to patients timeously leaving the ED once treatment is completed (1, 4). In July 2022 the Royal College of Emergency Medicine conducted a short survey which demonstrated that two-thirds of ED Clinical Leads were not confident that their organisation would safely be able to manage forthcoming winter pressures (5). The British Medical Association has reported that demand for care across all NHS England ED departments in June 2023 remained high with the total ED attendances standing at 2.22 million. Ongoing pressure on services and the backlog of care and chronic workforce shortages has meant that waiting times have increased to record highs. The number of ED patients waiting over 12 h from “admission to decision” was 26,531 in June 2023. This is 1.20 times higher than that seen in June 2022 (22,034) and 57 times as high as it was in June 2019 (416) (6). These difficulties were exacerbated with the advent of the COVID-19 pandemic.

Artificial Intelligence (AI)-supported triage and diagnosis has the potential to alleviate some of these difficulties. “Triage” in this study is defined as the process of assigning a degree of medical urgency to patients presenting to an ED, so that decisions can be made both on their order of treatment and on resource allocation. AI interventions hold much promise in healthcare. To date, AI-supported systems have been used to augment health practitioners in patient diagnosis and treatment activities for a wide range of diseases. However, although expanding rapidly, the real-world clinical implementation of diagnoses and triage AI-supported systems remains limited (7, 8). Moreover, users of these technologies hold varying attitudes towards their application in healthcare settings which can constrain their acceptance and utility (9). Thus, developers and implementers are well advised to consider attitudinal disconnects between different users in their acceptance for, usefulness of, and reservations about such technological adoption. The purpose of this study is to examine practitioner perspectives on the adoption of a proposed ED AI-supported triage diagnostic system. The study's objective is to understand and articulate the perspectives and attitudes of a small, discrete sample of NHS medical practitioners who may, in future, be tasked with using these technologies in the course of their duties. Understanding the attitudes and perceptions of users can assist in better guiding the development and deployment of proposed and future systems and in developing processes and policies that establish certain critical guardrails to implementation.

## 2. Background

Innovations using AI-supported technologies in healthcare are positioned to significantly affect the delivery and practice of medicine in the immediate and foreseeable future (10–12). The potential use of such technologies in healthcare is extensive and far-reaching (13). A recent acceleration in the deployment of technologies, supported by AI, machine learning, smart sensors, and big data analytics, has been seen to improve quality of care, decrease treatment cost, increase operational efficiency, expedite diagnoses and referral of treatable diseases, and to improve clinical outcomes (8, 14–19).

However, failure of AI-supported technologies can have serious, adverse consequences for both clinical outcomes and patient experiences which can erode public trust and can undermine trust in those healthcare institutions deploying them (20). Yet, despite an ongoing struggle to gain adoption in clinical settings, Hendry et al. found that even without a deep understanding of the technological capabilities of the system, practitioner trust can be built through experience, expert endorsement, and validation (21). Accordingly, the attitude and willingness of medical practitioners to trust and accept the technology remains an essential part in the successful implementation and uptake of automated healthcare systems such as DAISY (22–25).

Recent studies have explored AI-supported tools for healthcare purposes from the perspective of patients and consumers (26, 27). Moreover, research has been conducted on healthcare workers' attitudes towards, and confidence in, AI-supported systems, and on their impact on future healthcare, generally (28–31). However, this research is the first to capture the perceptions and attitudes of ED medical practitioners towards a triage AI-supported diagnostic system (under development). It is also the first study to raise important questions about the proposed system's benefit and the concerns of those practitioners who will be working alongside such a system in an NHS triage ED.

## 3. System description

Intended to support and augment human performance rather than replace human experts, AI-enabled medical technologies can execute certain tasks with greater consistency, speed, reliability, and reproducibility than human-agents (7). An example of such a proposed triage AI-supported system—the Diagnostic AI System for Robot-Assisted ED Triage (or “DAISY”)—is a collaborative effort undertaken between the NHS in England and the University of York. The project aims to develop an AI-supported system to automate the ED triage process. DAISY is a semi-autonomous, sociotechnical AI-supported system that directs patients through a triage pathway and captures both subjective and objective data. DAISY will enable a patient to input subjective information about their condition and will support the patient in using wirelessly connected medical devices to capture and record objective data (such as, blood pressure, pulse rate, temperature, and the respiratory rate). Patients are then guided back to a waiting area. Following data collection, DAISY utilises a complex, rule-based (“dAvInci” or Diagnostic Algorithm for Intelligent Clinical Intervention) algorithm devised by a medical expert practitioner to link patient characteristics, demographics, and symptoms, viewed through the patients' objective vital signs, to possible clinical states and to urgency and early treatment options. The DAISY system identifies potential patient maladies, suggests further investigations and patient referrals. The system returns possible or suggested outcomes given the patient data. Each of the information types (demographic, anatomic, subjective, and objective) are considered in parallel for efficient rule checking for maladies, such that the intersections of the resultant data type rules are possibilities. The algorithm returns a detailed report that contains a set of possible early diagnoses, as well as suggested continued investigations based on the objective and subjective data. These preliminary findings are then approved, amended, or rejected by the practitioner to facilitate the early stages of triage. The assessment with appropriate advisory information regarding a preliminary diagnosis and treatment plan is produced which the practitioner reviews and discusses with the patient.

Once operational, DAISY will expedite and direct the triage process by facilitating patient observations and providing practitioners with a preliminary patient report. We note here that while these potential diagnoses are useful for identifying additional tests or providing potential avenues for additional investigation, the benefit of the DAISY system is in the rapid categorisation of patients by severity, identification, and escalation of the critically unwell patients—and the generation of suggested investigation plans for subsequent approval by the practitioner. Practitioners can thereby streamline the early elements of the process to allow for additional treatment time and more effective resource management in critical cases. DAISY is not, however, intended to triage patients at the highest tier of triage illness—that is, those considered to need immediate life-saving intervention.

## 4. Significance of the study

The purpose of this study is to understand and explore the current attitudes and perceptions towards future DAISY development and implementation, and pathways and barriers to DAISY adoption. The study seeks to consider the implications of DAISY deployment in augmenting and assisting clinical diagnoses so that practitioner requirements are better incorporated into future iterations of DAISY design, and communications and training content can be developed to address perceived challenges and concerns about the system. The study seeks: first, to identify, broadly, practitioner attitudes and perceptions of DAISY adoption in a clinical setting; second, to explore certain user requirements and barriers to implement with a social, cultural, and ethical dimension, and third, to record perceived benefits and shortcomings of the DAISY system. In doing so, we progress the development of such systems in practical clinical settings by adding to the literature and identifying opportunities for further research.

## 5. Methodology

### 5.1. Participants and data analysis

Participants consisted of nine health practitioners representing a range of roles (hereafter, the “practitioners” or the “participants”). Participants were selected using the following inclusion criteria: that they were a clinical professional with emergency department experience through either current or previous roles. Only participants located in the United Kingdom treating NHS patients were included in the study. Participants were between 20 and 59 years (participants were asked to select their age-range by blocks of ten years), experience in emergency medicine ranged from 1 month to 22 years, and 5/9 of participants reported being “very” confident with using technology (2 reported moderate confidence, one reported limited confidence and one reported no confidence). All practitioners in the study were or had been employed at NHS hospitals across England and the sample consisted of five males and four females.

An interview schedule, drawing on questions from the Schema Action World Research Method (32, 33) and on the work describing social, legal, ethical, empathetic, and cultural (“SLEEC”) norms in autonomous-agent contexts (34), was developed to capture practitioner perspectives for automating the ED triage process (that is, deploying the DAISY system). The interview schedule is set out in Supplementary Table S2. In part one, participants were asked to describe the current (typical) process of ED triage, including clinical decision points. Questions centred around the role of past experiences and expectations in decision making processes, cultural considerations, and empathetic practice. In part two, participants were introduced to the functionality of the DAISY system and questions covered areas including its potential utility, influences on trust, and on the role of intuition and non-verbal cues in the patient-practitioner relationship and interaction.

Interviews were conducted on either MS Teams or via Zoom and lasted between 45 min to an hour. The in-depth interviews were recorded and transcribed verbatim. Additional notes recorded by the interviewers were included in the data documents. The interviews were automatically transcribed using the MS Teams function or manually and then revised by one of the authors for sense checking. The transcripts were then reviewed for accuracy and completeness by comparing the audio recordings with the transcripts. Line-by-line coding of transcripts was conducted, and data were organised into themes and sub-themes as set out in Supplementary Table S1. The coding was performed independently by two researchers. Coded transcripts were checked to include any novel or interesting responses that might not have been previously captured. During the analysis, members of the research team conducting the interviews were consulted regularly to review interpretations and discuss results. The data were thematically analysed using both inductive (generating insights from the data) and deductive (exploring data with SLEEC norms) approaches. An analysis was systematically applied to transcripts from the interviews to identify the themes, sub-themes, and insights described below.

The qualitative data analysis and interpretation research consisted of a process of data preparation, exploration, analysis, and interpretation. This comprised collecting the data; transcribing, organising, and cleaning the data; and coding, memoing (or capturing ideas about the data), and analysing the data. The data were then arranged into core themes and emergent sub-themes. Data were grouped into the following core broad themes (or overarching topics): trust, support and benefit; challenges and shortcomings; the social, legal, ethical, empathetic, and cultural aspects of adoption; and insights into process and practice. Developing sub-themes for “trust” included “reliability and trust in the system, and/or the process, and/or of the report, and/or of the diagnostic output”, “establishing patient trust”, and “trust in DAISY”. For “social, legal, ethical, empathetic, and cultural aspects” emerging sub-themes were “reliance on nonverbal and other physical cues and intuition”, “the role of empathy”, “the impact of DAISY on the practitioner-patient relationship”, “the role of reassurance and managing expectations”, “the importance of social skills”, “explainability and transparency of the system”, “privacy and sensitive information”, “equality and bias”, “cultural and social sensitivities”, “over- and under-reporting symptoms”, and “medical liability and duty of care”. Sub-themes in “process and practice” were the “report and preliminary diagnosis” and “current practice and workflow”. The core themes of “support and benefit” and “challenges and shortcomings” did not have specific sub-themes. It was within these broad themes and sub-themes that concepts and relationships between the data were organised and coded into an explanatory scheme, findings were reported upon, and a narrative discussion concluded.

The quantitative data analyses used the transcribed and cleaned interview scripts as inputs to Python text analysis tools:

• (i) Word Cloud (35) for statistical analyses of the most common vocabulary (words) used by the participants together (all scripts combined) and individually, and
• (ii) sentiment analysis using a transformers (36) pipeline with the bhadresh-savani/distilbertbase-uncased-emotion (37) emotional analysis large-language model (LLM) to analyse the scripts together (all scripts) and individually.

WordCloud (i) identified the 25 most frequently used words (excluding stop words, “yeah”, “patient” and “patients”) in:

• The text from all participants (all-text),
• The text from each participant separately (p1-text, p2-text, … p9-text)
• The text from all participants which referred to DAISY (DAISY-text),
• The text from each participant separately which referred to DAISY (p1-DAISY, p2-DAISY, … p9-DAISY).

This highlights the important aspects in the vocabulary of the interviews generally and with respect to DAISY.

The emotional (sentiment) analysis (ii) used a LLM to label the participants' answers (or query text) with human emotions and sensitivities. The distilbert-base-uncased-emotion model uses the labels {joy,love,surprise,anger,fear}. These can be converted to emotion scores {joy=+1,love=+1,surprise = 0,anger = -1,fear = -1}to represent positive (+1), neutral (0) and negative (−1) emotions. Statistical analyses of the scores were obtained for:

• The text from all participants (all-text),
• The text from each participant separately (p1-text, p2-text, … p9-text)
• The text from all participants which referred to DAISY (DAISY-text),
• The text from each participant separately which referred to DAISY (p1-DAISY, p2-DAISY, … p9-DAISY).

This highlights whether participants are positive, neutral, or negative regarding the topic under discussion.

For the statistical analyses, the scripts were subdivided into sections (of fewer than 500 words each) as the emotional analysis LLM model requires inputs not exceeding 500 words. The texts were subdivided as naturally as possible, keeping similar text together and splitting on context changes as much as possible to prevent subjectivity of analyses. The LLM labelled each section of each participant's responses which were then scored. We calculated a sample mean score *x* for (all-text) and (DAISY-text) by summing the section scores and counting the number of sections. The sample mean score assessed the emotional positivity of the text.

The word statistical analyses and emotion analyses used the text from part 1 and part 2 combined (all-text) and then the text from part 2 where the participant talked specifically about DAISY (DAISY-text).

### 5.2. Ethics

This study was reviewed and approved by the University of Southampton Research Ethics Committee in June 2022 (Ergo ID: 72301). In addition, permissions from respective institutional gatekeepers were obtained to access potential participants from the NHS as required. All participants were asked verbally to consent to the research and agreed to the recording of their interview. All personal and institutional identifying data were removed from the interview transcripts before coding and analysis.

## 6. Results

### 6.1. Participants

The inductive analysis grouped the data into core themes: trust; support and benefit; challenges and shortcomings; the role of empathy and the social, legal, ethical, and cultural aspects of adoption; and process and current practice. In relation to current practice, variability in the ED triage process between hospitals was apparent which would need to be considered if the system was more widely rolled out across the UK or elsewhere.

**Figure 1:**
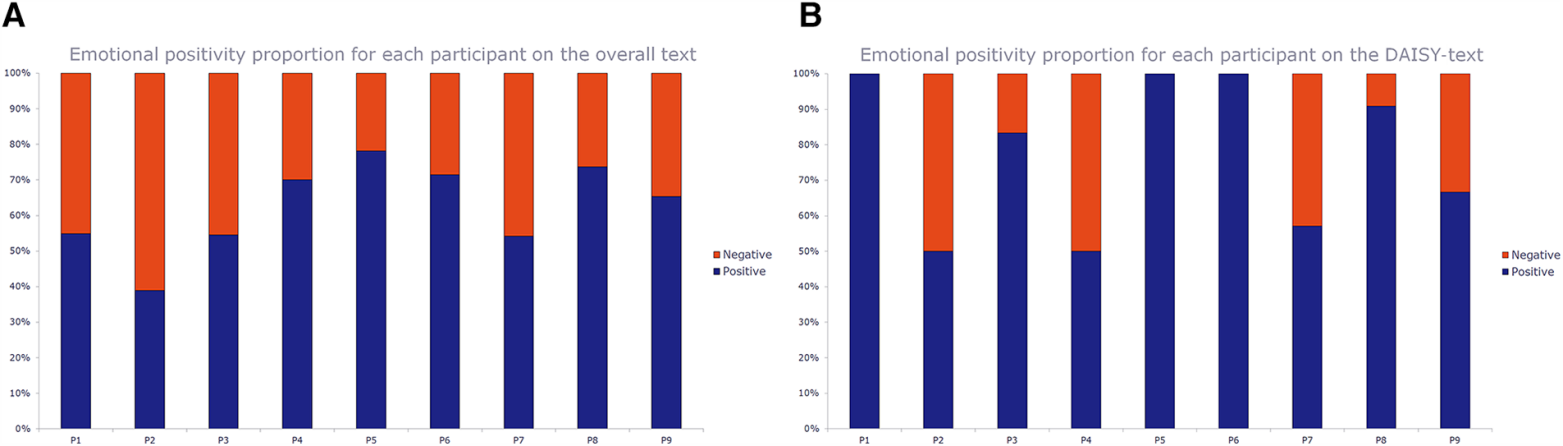
Charts of the emotional positivity proportion (positive vs negative) for each participant (n=9) on the overall (all-text) text (left) and the DAISY-text (right). The bars show the proportion of positive text sections vs negative text sections for each participant (P1-P9) using the data in Table 1.

Across all nine interviews, DAISY was, generally, favourably perceived and considered of benefit to the triage process as can be seen in the chart below showing the proportions of positive vs. negative sections in the overall text and DAISY text for each participant (Figure 1). The statistical analyses of the emotion scores (see Table 1) showed $\bar{x}$  = *0*.*55* (*stdev* = *0*.*43*) for DAISY text compared to $\bar{x}$  = *0*.*24* (*stdev* = *0*.*25*) for all text indicating that the participants speak more positively about DAISY than ED practice in general (although P4 was less positive about DAISY than overall), and that there is more of a spread of emotions when talking about DAISY than generally. Compared to the current ED system, P4 was concerned at DAISY missing non-verbal cues, patient perceptions of not seeing a human, and the availability of explanations for diagnoses of patients. No participants had a negative emotion score when talking about DAISY whereas participant 2 had an overall negative score $\bar{x}$  = −*0*.*22*. The overall perception of the practitioners was that DAISY could play a supportive and assistive role in diagnosis and that they would welcome integrating DAISY into the triage diagnostic process. This with the caveat that certain safeguards and protections be implemented. Concerns were identified. They included the inability to verify the algorithm for misdiagnosis and to share control in decision-making outcomes. Trust and reliability in the system and the diagnostic output were identified as barriers to adoption, particularly in the early stages of implementation, as were concerns around the quality and integrity of the input data. “Trust” is a common word in the combined DAISY-text and for two participants talking about DAISY, with “privacy” a common word for one participant talking about DAISY. The expectation expressed by participants was to see a percentage match between practitioners and DAISY diagnoses (with a yet to be determined or unknown threshold for acceptance) before trust in the system could be established and there was consensus that gaining trust would be an evolving and dynamic process.

**Table 1:** Emotional positivity score for each participant (n=9) on the overall (all-text) text and the DAISY-text, along with the number of (up to 500-word) sections scoring positively (Count Pos) and negatively (Count Neg) in the respective texts. There was one section for P1 that scored neutral and one section for P6 that scored neutral - both in all-text (the neutral counts are omitted for space). From these, we can calculate the sample means and standard deviations of the emotional positivity.

	All text			DAISY text		
Participant	Positivity	Count pos	Count neg	Positivity	Count pos	Count neg
P1	0.09	17	14	1	10	0
P2	−0.22	7	11	0	6	6
P3	0.09	6	5	0.67	5	1
P4	0.40	7	3	0	2	2
P5	0.56	25	7	1	4	0
P6	0.41	20	8	1	5	0
P7	0.08	13	11	0.14	4	3
P8	0.47	14	5	0.82	10	1
P9	0.31	17	9	0.33	6	3
Total		126	73		52	16
Sample mean	0.24			0.55		
St Dev	0.25			0.43		

Regardless of job role, practitioners stated the importance of non-verbal cues and intuition when treating patients and the role of local knowledge (for example, that of known drug offenders). Concern was expressed as to how these would be accounted for by the DAISY system. The ability to accommodate cultural and social sensitivities and to act empathetically were indicated as significant. That patients seek reassurance was stated as one of the primary and important outcomes of patient-practitioner interactions and the DAISY system was seen as potentially advantageous and value-adding as it freed up practitioner time from routine tasks which allowed them to spend more time with patients. Other added value included the standardisation of the quality of triage reports and consistency in reporting. An interesting observation was made, that of ascertaining the likelihood of patients to disclose sources of (potentially embarrassing or sensitive) injury or domestic violence to a non-human system. It was believed that in certain instances patients might be more willing to disclose information that is perceived as sensitive or shameful to a system incapable of human moral judgement or criticism. Other positive cultural implications were expressed, including the potential for DAISY to work in any language, which has the potential to provide more accurate information (assuming translation back to English was accurate) to assist the diagnostic process for non-English speakers.

Charts of the emotional positivity proportion (positive vs. negative) for each participant (*n* = 9) on the overall (all-text) text (left) and the DAISY-text (right). The bars show the proportion of positive text sections vs. negative text sections for each participant (P1-P9) using the data in the Table below.

Table of the emotional positivity score for each participant (*n* = 9) on the overall (all-text) text and the DAISY-text, along with the number of (up to 500-word) sections scoring positively (Count Pos) and negatively (Count Neg) in the respective texts. There was one section for P1 that scored neutral and one section for P6 that scored neutral—both in all-text (the neutral counts are omitted for space). From these, we can calculate the sample means and standard deviations of the emotional positivity.

### 6.2. Specific perspectives on DAISY adoption

**Figure 2:**
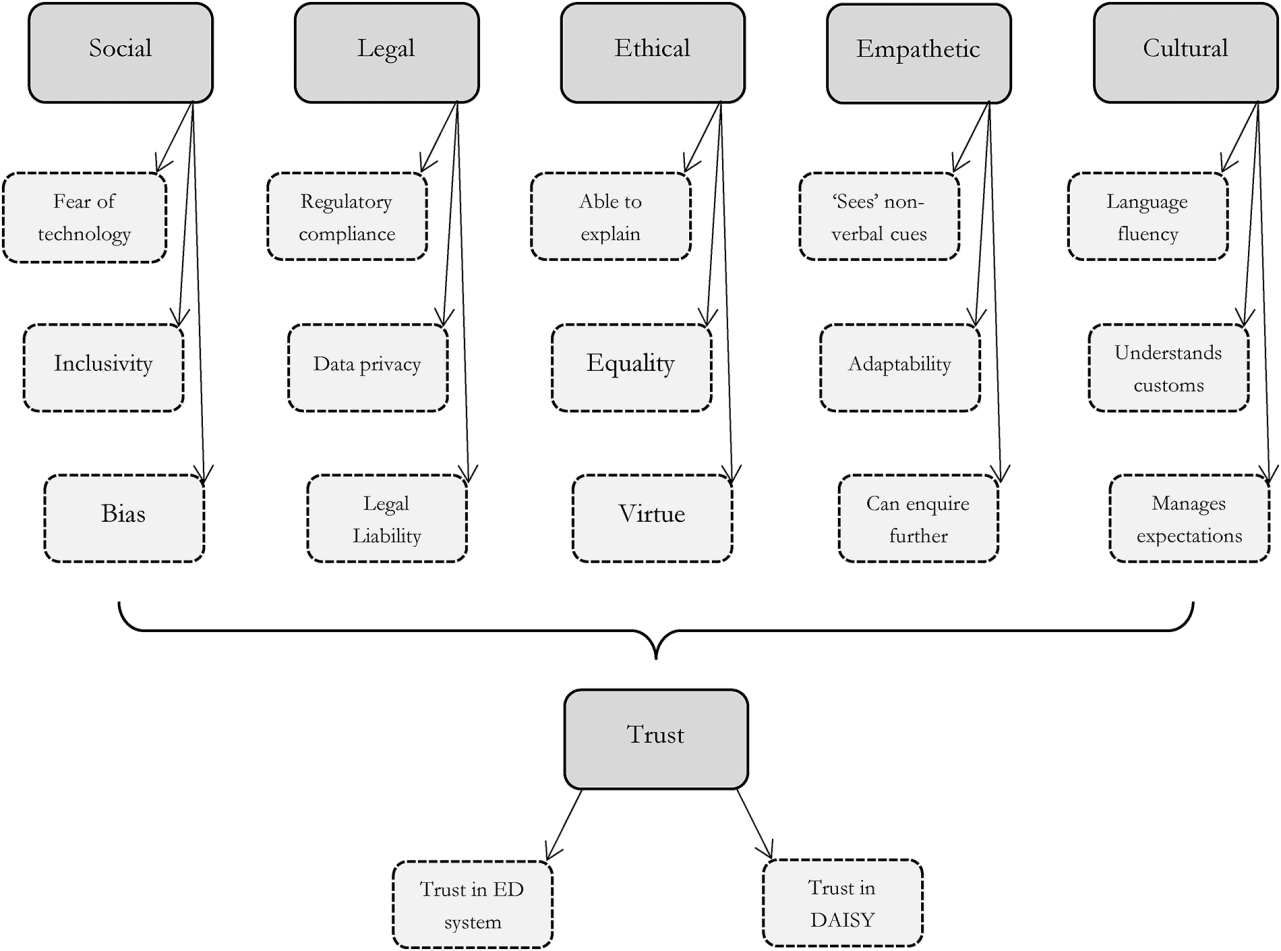
Overview of the main themes and their linked sub-themes (shown by dashed outlines) that were elicited from the participant interviews. Trust is the over-arching theme elicited from the interviews.

The data revealed that although participants supported the adoption of the DAISY system, concerns were identified. Appended Supplementary Table S1 sets out participant comments and Supplementary Table S3 specifically indicates social, legal, ethical, and cultural concerns. Comments are arranged according to subthemes: that is, support and benefit; concerns and shortcomings in DAISY adoption; trust; explainability and transparency; reliance on non-verbal cues and intuition; empathy and social and cultural adaption and sensitivities; reliance on reassurance; privacy, data protection, and data security; and additional observations. Certain comments and immediate concerns informed wider themes, such as the implications and possible erosion of the practitioner-patient relationship or the collection of personal data and the impact on data quality, privacy, and security. Many concerns expressed are not isolated anomalies or particular to DAISY uptake, but are recurring themes in digital technology adoption, practice, and research (38). The main themes are illustrated in Figure 2, along with each theme's key sub-themes.

#### 6.2.1. Benefits and support

Participants were overall supportive, but cautious, of DAISY triage adoption. Benefits cited were a reduction in patient wait times in the ED, assistance in the streamlining of the triage process, support in calling for appropriate diagnostics and for further patient examination, and the identification of those very unwell and requiring more immediate and urgent attention. Common words in the DAISY text include “good idea”, “help”, helpful, “easier”, “value”, and “accurate”.

Reducing time in the ED was seen to be of huge benefit. “Time” is a common word throughout the interviews. Participants had “quicker”, “speed”, “delay” and “delays” as common words when discussing DAISY but not overall. Participants stated that: “*it could speed things up*” and “*it might prevent people from sitting in the waiting room*” and “[if preliminary work] *can be done early on, that can drastically reduce the amount of time that patients are kept within the department”*. Further observations were that: “*I think that* [DAISY] *would be beneficial. Patients would be seen by something quicker and would be able to triage and* [establish] *red flags for those [patients] who are really unwell, and then they could be alerted and put to the top of the list”.* One participant stated: “*I think* [DAISY] *would be quite beneficial. …I do not think DAISY in itself needs to be replacing anyone but* [I] *feel it can contribute to streamlining what we do”*.

The attitude of practitioners was that even basic assistance can be very useful. A participant stated: “*I think its use is in reducing the time I spend during the consultation trying to take history”.* Another participant went further regarding clinical decision-making: “[Although] *DAISY is not going to take a full, thorough history, but if it is able to take just the basics for me and I can then touch up on what is missing from that, that would reduce the length of time that I spend with the patient and if the investigations get ordered earlier, much earlier, then I can try and tie up that entire consultation sooner. So, I have all the evidence I need, all the investigations I would have ordered, I have the history with me, I have my clinical examination findings and I can then combine all of that into what I think is going on and decide on what management the patient needs or whether they can go back home or whether they might need to be admitted”*.

#### 6.2.2. Concerns and shortcomings

The idea that the practice of medicine is both an art and a science was identified by the participants. DAISY's inability to practise medicine as an art is viewed as a potential shortcoming of the system. The reliability of the algorithm and the potential loss of practitioner oversight were also identified as of concern.

Participants stated that in making a diagnosis: *“ you use all your senses and so* [how can you] *design a* [triage] *system that does not have a sense. And the answer is very simple. As you know, as a junior doctor, I did not use my senses. DAISY can never be* [that]*. I do not know, maybe somebody will design that*. [DAISY] *is not a consultant who uses their senses and smells the patient and feels”*.

Context and location appropriateness were identified as potential shortcomings: *“The trial data must be location specific…. There may be cultural elements to it. If we introduced* [DAISY] *in a different country, we would get different evidence*…[for example] *reported health symptoms may be slightly different”*.

On detecting subtleties in the information patients provide such as regarding abuse and addiction: “I *think an automated system might have a downfall in those sorts of cases*”. Patients may just “*say I could not tell my whole story, or their story could potentially be misinterpreted*” to (subtly) indicate such circumstances. Participants highlighted the need for human care in the diagnosis and treatment stating that patients like “*to feel like they have been seen*”. The loss of human connection was cited as a disadvantage: “*I feel like you cannot really build a rapport with a robot*”.

While most practitioners were positive, certain participants were dismissive of the use of DAISY stating: “*I don't think I would be a fan of the robot. I would want* [to see/be seen by] *a person*”*.* Other misgivings were: “*People* [who are not] *very computer literate …would never have a clue how to use it”,* and outright refusal to use the system; “*I would not use this, it would not be workable*”.

Further shortcomings were identified. Practitioners expressed concern regarding the cost and viability of introducing DAISY to the healthcare system, and the cost to update and maintain the system. A participant stated: “*.. in terms of cost, I think that might be one of the major things with incorporating DAISY into healthcare and in terms of whether its staff members need to be taught how to use it or navigate around it and the maintenance cost. So … the benefit of DAISY and what it brings into the ED triaging system, needs to outweigh the cost of having it, purchasing it, and maintaining it in the long term. That would be one of the key points to address before roll-out to most healthcare facilities”.* Participants also identified that certain patients would be unwilling or reluctant to change*, “especially older patients some of whom are still not happy using telemedicine”*.

#### 6.2.3. Trust

Participants emphasised the need for the system to be trustworthy in practice and trusted both by the practitioner and the patient. Trust is a critical factor influencing the utility and wide-spread adoption of the system. Certain participants expressed hesitancy in uptake until such time as credibility, reliability, and regulatory approval of the system and the underlying algorithm could be demonstrated: “*I don't think you would be able to trust everything at face value, at least not to begin with”.* Further consideration regarding trust: “*To trust a system like this I would need to prove that it works and does not miss anything. I think if it was implemented at the hospital where I work today, I would always double check everything it did* [if] *only because it has not been used before*”. Reservations were expressed by participants: “*I do have reservations. I would have to see trial data to know how good the algorithm is …. There are nuances to this* [diagnosing]*”.* Another stated: “*I would need to know that* [DAISY] *has been properly tested”*.

Participants were also concerned that DAISY might not be reliably or securely implemented resulting in reputational vulnerability to both the practitioner and the system itself. One stated: “*If* [trust] *is diminished, then it is very hard to come back from that”*.

#### 6.2.4. Explainability and transparency

Explainability and transparency were recognised as important factors in the system's adoption. A participant stated: “*It is important to be fully transparent”.* Participants wanted to know the basis upon which determinations and underlying assumptions are made in the diagnosis and the reasons for doing so. They stated: “*I would like to know why a diagnosis was made”* and “*I would want to know this* [so as to know whether] *to do further investigations to confirm or deny* [the diagnosis]*”.* Minimally, the information required by most patients is “*what the diagnosis is, any treatment they need, and probably the duration of symptoms”.* Moreover, the nurse practitioner suggested that more detailed descriptions are needed: “*You need a plan, you have to say we are taking these steps. And if certain things do not work, then you must see your GP, a therapist, and so on. So, if you do not have an immediate diagnosis, you need to plan for what happens next”*.

#### 6.2.5. On the reliance on non-verbal cues and intuition

Practitioners emphasised the role of, and reliance on, clinical expertise and intuition. The perception was that intuition is not easily replicable by DAISY. The richness and diversity of visible cues, and the use of practitioner intuition were identified as valuable elements in patient diagnosis. A participant stated: *“I think with a lot of healthcare there are certain things that you could miss if you just go through an algorithmic way of dealing with things. There is a lot of what is called “gut feeling” involved. When someone presents to the robot to do observations that is fine, but they are just numerical values. Sometimes you just look at the person and you can tell they are really sick”.* Importantly, a diagnosis is made within the context of “ *what the patient tells us”* as well as within “*the background of what I can see, what I can observe, what I can sense”*.

One participant commented: “*A lot of the time we will get people saying that they just do not feel right, so then it is up to us to pick out what they mean by that* [it takes] *a lot of observational skills to figure out what is going on”.* Another participant remarked: *“There are times when I think they do not look right but* [I] *cannot put a finger on it. But I know something is wrong…* [I] *suppose* [this is] *the sign of years of experience”*.

Regarding the reliance on intuition and “gut feelings” when there is reason to believe that something does not “look” right, a participant said: “*I have a feeling, so that means that on the patient's* [account/observations]*… everything looks OK, but I just do not like the look of that patient. You know, sometimes nothing was* [indicating] *that, I just do not like what I am looking at. Even though all the numbers are fine.”*

Following this, a participant remarked: *“DAISY* [will not have discretion] *and will simply* [accept that] *the patient says they are fine.* [As] *the numbers say they are fine, so they are fine”.* Further emphasising the importance of, and reliance on, non-verbal cues, a participant stated: “*You can tell from a patient's body language when they come in… there are a lot of non-verbal cues.. the way they interact* [needs to be] *factored in”.* Accordingly, *“you use intuition …you can see people* [who] *look ghastly. You know, they look a bit grey or just when they are particularly quiet, it is the quiet ones that are* [often] *a bit more poorly”*.

#### 6.2.6. On empathy, and social and cultural adaption and sensitivity

The ability to express empathy and to act and be treated with dignity was identified as a key component in the practitioner-patient interaction. A participant stated that the practice of “*empathy changes from patient to patient*” and that “[I] *adapt the way I respond to a patient depending on the patient within a situation”*.

Empathy was repeatedly emphasised in the data as critical to good patient care and practice. A participant opined: “*A patient* [and] *any human wants to feel appreciated. You know, you walk into the room* [and say] *Hello. How are you? And how are you today? … I let the patient feel as though I am listening to them … And then to be seen very promptly and not to be kept waiting, and if they are going to be kept waiting to be given information regularly* [about the wait]*”*.

Crucially, one participant felt that DAISY would fall short in this regard stating that: “*There is a human factor that is removed by using DAISY”.* On the importance of expressing empathy and sensitivity to patients and their unique requirements, the following was expressed: “*It is listening to the patient… to understand why they are* [seeing you]*”* and expressing that “*If* [the patient] *is not comfortable, we can stop, and I will get another clinician to see* [them]*”*.

On a positive note, a participant identified that DAISY may assist in overcoming language barriers: “*We get lots of patients that do not speak the language that we speak. So, we struggle to communicate with them… if DAISY could* [communicate] *in* [various] *languages, and even when they have interacted with the robot in a particular language,* [the practitioner] *could get a report in English* [this will assist us]*.”* Another participant added: “*if DAISY had different language settings that would be really useful*”.

Participants also observed that language and disabilities might be a barrier to adoption. DAISY may be confronted with patients that have difficulties in articulating and expressing themselves and their conditions. Questions of inclusion and diversity were posed. A participant commented: “*We have language barriers, … English is not necessarily a patient's first language, so we might have to explain things differently”.* Also, *“We have different dialects and accents, and some people have much stronger accents… it might be more difficult for DAISY to interpret what they are trying to say. So, it might not work as well or as intended with specific demographics”.* Moreover, “[with regard to] *patients with hearing aids or who are a bit hard of hearing, DAISY may not necessarily be well tuned to this if* [such patients] *are not able to properly articulate what they want to say”*.

Nevertheless, the observation was made that there is merit in the DAISY system even if it cannot now accommodate all patients: “*But, if DAISY can do a good job with your average patient.. and then we can delegate the others to a* [human] *healthcare professional that would still be an advantage because it opens the room”.* Another participant added: “*We will still need the triage nurse for the patients that are not suitable for DAISY, but it might halve the workload for the triage nurse if we have sufficient robots*”.

#### 6.2.7. On reassurance

The role of practitioners in reassuring patients, that is, putting their minds at rest and providing comfort, was seen to be significant. Many participants believed that reassurance is an essential part of the triage process and that values such as honesty and transparency allay fears and instil patient confidence.

Participants stated that: *“You know that the biggest intervention that we give our patient is reassurance. Somebody will attend to you* [and] *you will sort the problem out. That is ….we say we are going to sort the problem out or we will try our best to solve the problem”.* The value to patients of human connection underpinned by assurance and practitioner honesty was reiterated: “*Just comforting and reassuring the patient is important because sometimes we do not know the answer”* and “*I think we also have to be honest when we do not know the answer”*.

#### 6.2.8. On privacy, data protection, and data security

The DAISY process must respect privacy—that is, preclude others from intrusion into a patient's personal space. As DAISY collects and generates personal, often sensitive, health and demographic information, important considerations of data protection, data quality, and data safety were identified as a concern. The disclosure of sensitive information and the adverse consequences of data breaches concerned participants.

Participants stated regarding disclosure of personal information and the perceived comfort in interacting with a nonhuman agent: “*….DAISY will go objectively and not care what the patient looks like,* [concluding] *this does not sound right. The other advantage in a robot.. is that some questions we do not ask our patients, DAISY will….. And to be honest,* [the patient] *might feel more comfortable responding to a robot about domestic abuse or something embarrassing”*.

Regarding concerns about privacy in the current process: “*If it is just a curtain between you and the next person, I think we have to be realistic that… drawing a curtain round a patient does not magically block out all the sound”* and “*You know there are certain things people will not necessarily say at the front desk, but if it is in the privacy of the room they might say”*.

Concerns regarding data protection were identified: *“One aspect that needs to be looked after would be the data safety and confidentiality… I assume that DAISY would be connected to* [a] *cloud back-end* [and] *that it needs to be ensured that it is secure and safe because if there were any data breach, that would lead to… a data leak and all patient data could be compromised”*.

#### 6.2.9. Additional observations

DAISY was identified as potentially valuable as a back-up or second resource. A participant stated: “*I do not know if DAISY would be able to cross check medications with nurses, because sometimes they* [may] *want to administer medications that need to be cross checked by two healthcare professionals just to make sure that everything is correct… Could DAISY be the other of those healthcare professionals?*”. It was also suggested that preliminary diagnoses by the system might lead to undue influence and support confirmation bias, that is, once a DAISY diagnosis is made, only corroborative patient signs and symptoms are looked for by the practitioner.

General questions were posed about how a system malfunction might be detected and where redundancies in the system might be. Greater integration into the existing process is also required: “*that might take a bit more effort to integrate, but I was wondering whether history gathering, and diagnostic formulation can be integrated into the back-end software that is run by the hospital itself, so information gathered by DAISY gets transcribed into a mini consultation—or clinical sheet—that would then be produced*”. Comments about additional or extended uses for DAISY (or other AI-supported systems) include: “*Could DAISY be used to chaperone* [patients] *to a room or as a chaperone while being examined?”*.

Many participants offered responsiveness and the ability to sound and act with human-like qualities as helpful DAISY attributes. A participant stated: ‘*I think the main issue would be how proficient DAISY is in conversing or carrying out a conversation with someone. So, if you think of the early days, for example, when SIRI was first introduced, initially it sounded quite robotic and it was not able to respond* [well]*, other than to a small set of fixed responses* [questions/requests] *and things to reply to. Anything else that you tried to say to it, it would come out with an error or just tell you “I don't know what you're saying”. So, if DAISY ends up being similar, I can see why it would be difficult for patients to accept it. But if we can work on improving the tone and modulation of DAISY ’s voice and make it sound more human-like and natural,* [the system would be more readily accepted]’. We also require a ‘*repertoire of responses’* and to *‘integrate this… so that it will be able to carry out a proper conversation with someone*’. It was asked whether DAISY would only ask closed questions or would be capable of probing for further and detailed enquiry, and whether the questioning would be variable and input dependent.

The appearance of DAISY was cited as significant. Participants were curious about what the system would look like, whether the system would comprise simply a touch screen, and the degree of sophistication of DAISY. It was observed that the use of colours and branding would impact perceptions and usability. That DAISY look, sound, and act personably was important, with one participant commenting: “*maybe make it look a bit more friendly”*. It was also stated that different demographics, for example the elderly or very young, might find the system difficult to operate, inaccessible, unapproachable, or frightening.

## 7. Discussion: social, legal, ethical, empathetic, and cultural barriers and considerations

Our study demonstrates that trust in the system is a significant driver of use and a potential barrier to adoption. Overwhelmingly it was seen that trust takes time to establish. This is consistent with similarly reported findings in the literature (22). Generally, practitioners who participated in the study indicated a benefit to using the system. The emotions labelled by the LLM were more positive when the participants discussed DAISY compared to discussing ED practices in general. The main benefits were in the reduction of ED wait times and increasing consistency in the triage process. This was very well supported by the participants. “Time” was one of the most common words used throughout the interviews. Other positive outcomes included improved diagnostics, increased efficiency, improved access to and quality of care, and increased objectivity.

Identified risks and challenges create the opportunity for further research, particularly from an end-user (patient) perspective. A recurring theme in this study was the role of empathy. The importance of this in practitioner-patient relationships is well known (39), and has been shown to reduce pain and anxiety and not receiving “empathic care” (even if clinically appropriate care has been provided) can leave patients dissatisfied and in some cases traumatised (40, 41). In our study, while practitioners expressed concern that DAISY could reduce the experience of empathy in practitioner-patient relationships, it is necessary to better understand the patient experience of empathy when interacting with AI-supported healthcare technologies, both hypothetically and in practice. For example, it might be that expectations around empathy are lower in a “robot” scenario, coupled with a practitioner having more time to spend at the diagnosis consultation (rather than triage) stage of the process, thereby enhancing perceptions of empathy using DAISY and similar systems.

The data demonstrate that further critical and relevant assessment is required to better understand the social, legal, ethical, and cultural elements and implications of real-world DAISY implementation. There is apprehension around patients' ability to use the technology (particularly the elderly) and thus the likelihood for exclusion of those patients who have limited technological competence, are unwilling or unable to use the system, or lack confidence in using what may be perceived to be a complicated system. Moreover, questions of inclusivity, diversity, and equitable access to the technology were asked, for instance, who will have access to the technology? and will the cost to implement and maintain the system preclude access and availability?

It was found that accommodating various languages and accents, and cultural and customary sensitivities, will support use, and enhance patient interaction and overall experience, such as calling for a chaperone where appropriate or conversing in a language most comfortable for the patient. Sociotechnical systems of this kind exist within a social, ethical, and cultural context and operate at proximity to the patient, typically within their personal space (34). It is thus beneficial for the patient if the system presents cultural and social nuance in its interaction with the patient and does so in a way that mirrors human empathy, so that the interaction and experience can promote human wellbeing. It was cited as important to include non-verbal patient cues in making observations about patient health and to make the system appropriately responsive and adaptive to individual cases. Embedding virtues of care such as patience, tolerance, and compassion into the patient interaction was seen to be helpful. This includes, for example, displaying cultural sensitivity by addressing the patient in a preferred manner, acting politely, for example, by saying please and thank you when requesting that a task be performed, and acting to prevent patient discomfort where sensitive information is obtained by appropriately probing and questioning individual patients. As with human interactions, the system will from time to time be placed in a position where it will be required to practically resolve potential ethical dilemmas and adapt for cultural nuances. The system may be faced with decision-making requiring a degree of normative (or ethical or cultural) choice, for example, confronting the potential hypothetical scenario requiring it to trade-off, for example, respect for human autonomy (by always following a patient's instruction) and the prevention of harm or injury (by not following a patient's instruction for reasons of safety). What must be ascertained in these instances is to determine whether, in the circumstances, such normative AI decision-making ought to be taken by the system itself, involve human stakeholder engagement and input, or be left or delegated to human support. This will likely be informed by various factors including, amongst others, the context, potential risk, impact, and severity of the normative decision-making outcome.

An important consideration for future iterations of DAISY is the ability to provide explanations to the practitioner and patient. Explainability and transparency were identified as critical, with practitioners wanting to know *how* a diagnosis was suggested and *why* a particular diagnosis was suggested to the exclusion of others. Workflow issues remain a concern. Participants felt strongly that nurses should not have to troubleshoot or be responsible to assist patients in using DAISY. The need for health records to be digitised and shared across the health system was highlighted as important. As was greater flexibility and patient empowerment in healthcare, with the suggestion that in future patients might wish to use health technology remotely (or from the benefit of their home) before coming into the ED. It is also critical to manage expectations, both for practitioners and patients, by clearly indicating the system's capabilities and limitations.

All practitioners were reluctant to use the system without first satisfying themselves that the system and its output were both safe to use and accurate. Regulatory issues identified include concerns around the safety and efficacy of the algorithm (including the testing, validation, and certification or approval of the system). Concerns raised were whether there is sufficient regulatory oversight mechanisms in place, and whether, for example, the algorithmic quality can be assured, updates managed, and the algorithm adjusted for locations. Further challenges identified by participants are misdiagnosis and scepticism around the accuracy of the diagnosis and the report and the potential for patient values to be misrepresented. The ability of DAISY to call for patient history and further information also speaks to the importance of resilience and flexibility in the system. Potential shortcomings also included the quality, accuracy, and credibility of the input data and around the processing of, and access to, sensitive personal data, data breaches, and the physical privacy of the patient interaction, more generally. Greater clarification was required around responsibility, accountability, and liability. Questions were raised about who would be responsible or accountable for what and at which stage of the process. Moreover, the moral responsibility and legal liability of the practitioner should a diagnosis be incorrect or missed were raised as concerns. If left unaddressed, these factors could largely impede the system's uptake and use.

The transformative role of autonomous agents in healthcare applications brings about specific medico-legal and ethical concerns. This stands to implicate the traditional practitioner-patient relationship: one built on a long tradition of ethical norms, professional guidelines, and legal regulation. Applications—such as the use of AI in diagnosis, treatment, and triage will change the practitioner-patient relationship in clinical practice with the medical practitioner becoming increasingly reliant on tools and algorithms to inform diagnosis and treatment modalities (42). The challenge is that the practitioner-patient experience—one that is both effective and ethical—is now subject to an additional layer of progressive technological complexity (43). This also informs issues of informed consent and what such consent would mean in AI-enabled healthcare applications and of shifting notions of patient autonomy (44, 45). Would it be possible, in future, for patients to refuse AI-supported care and be permitted to demand human practitioner diagnosis and care? Edmond et al., for example, describe a “RoboDoc” or robots-as-doctors and the arising ethical implications such applications would bring (43). Of significance is the on-going requirement to fully understand both patient and medical practitioner needs, and to identify moral and ethical principles that shape this changing healthcare process. This is an area that remains highly relevant and requires further research.

## 8. Strengths and limitations

Limitations include a small sample size and the potential for biases inherent in the selection of participants. The interview sample represented only a limited subset of NHS practitioners drawn from a small selection of hospitals, including the NHS York and Scarborough Trust, John Radcliffe Hospital Oxford, Sheffield, and Southampton General Hospital. This limited selection of UK hospitals may affect the generalisability of the findings across geographical settings and worldwide. A further limitation is that the participants were asked to imagine a prototype AI-supported agent working alongside the practitioner. The interviewers described the functionality and envisaged workflow of the proposed DAISY system in detail. In addition, each participant was only interviewed once, and no longitudinal data were collected which would have allowed for the exploration of emerging issues in more depth and the opportunity to understand changes in perceptions and experiences over time.

However, the research provides a systematic search for meaning in the contextually-laden, subjective, and richly detailed data collected from the health practitioners. Our findings also provide a strong base for empirical research to further understand challenges and benefits to DAISY and other technology-based healthcare development and implementation. Some of these findings are generalisable and can be translated to inform and support the development of other AI-enabled healthcare applications and to understand the implications of their adoption. Because of the limited scope and size of the study, further extensive and diverse studies are crucial to validate these findings.

## 9. Conclusions and future directions

Notwithstanding identified risks and challenges, the findings of this study demonstrate that practitioner perspectives of DAISY adoption are overall positive and supportive of implementation. AI-supported technologies should be seen to augment and assist, rather than replace, human medical practitioners. In line with previous research, AI interaction in medicine should expand and aid the efficiency and effectiveness of human interaction and care pathways (12). Practitioner insights have proved invaluable for understanding end-user perspectives, which will be used practically to refine the prototype DAISY system for pilot testing in a custom-built testbed. In addition, an interactive online survey (using videos of the working prototype) is in development to capture patient perspectives of the system.

Triaged patients are frequently in a vulnerable state, and the process requires an awareness and sensitivity to the numerous socio-cultural dimensions of the triage interaction, and to the challenges presented by diverse populations with different needs and ways of acting, relating, and narrating. Recommendations for future research and development include better integrating existing norms and practices into DAISY adoption, capturing both technical and non-technical practitioner requirements, and embedding social, legal, ethical, empathetic, and cultural aspects into the system. Against this background, insight from practitioners can be used to implement incremental changes to avoid potential harm to patients and to ensure that the system is legally, ethically, socially, and culturally sensitive or compliant (for instance, it respects data privacy, is robust, secure, and safe, and acts socially and culturally appropriately). Specifically, future directions could incorporate cultural and customary sensitivities into the system, address cultural and language barriers and accessibility, find ways of better managing patient and practitioner expectations and workflows, and strengthen AI policy guidance. There is much opportunity for exploration into the social, legal, ethical, and cultural-responsibility implications of AI adoption in healthcare settings, for greater practical and contextual integration, and to establish how to build these socio-cultural aspects into the AI lifecycle more generally.

A further area of expansion is to better integrate the system into current clinical workflow processes within the hospital and to educate and involve medical personnel in the adoption process. AI integration in triage contexts—and indeed in other medical settings—requires considerable buy-in and collaboration with medical staff. Systems should be developed to support human practitioner autonomy and decision-making across the entire triage workflow with the role of the human-system interaction and accountability clearly demarcated. In addition, the implications of DAISY as a “partner” or “teammate” working alongside the practitioner and how to facilitate this requires investigation.

As this study was based on a relatively small and localised sample group, a clear call exists for further study to validate and supplement these findings. Moreover, additional research is needed to establish the association between DAISY and patient satisfaction and to help researchers, developers, and implementers find solutions to the specific concerns and barriers identified in this study. These challenges to AI-supported adoption are shared across medical settings, application types, and geographical locations.

## Data Availability

The raw data supporting the conclusions of this article will be made available by the authors, without undue reservation.
