# Supplementary material for: Medical practitioner perspectives on AI in emergency triage
Source: Front Digit Health. 2023 Dec 6;5:1297073. doi: 10.3389/fdgth.2023.1297073 (PMC10731272; doi:10.3389/fdgth.2023.1297073)
Supplement: Supplementary file 1 [file Table1.docx]

**Supplementary Material, Table 1: Participants comments**

| Themes | Practitioners’ comments |
| --- | --- |
| Trust: Reliability and trust in the system/process and of the report/diagnostic output  Establishing patient trust and trust in DAISY | Patients would like ‘*to feel like they have been seen*’    ‘*I think most I trust - the vast majority of my patients tell me the truth*’.    ‘*We must trust most patients and what they say to us’*.    With patients and over time ‘*you begin to notice when people are not necessarily fabricating things completely but enhancing certain parts of the story to get what they want*’    ‘*Always remember the patient doesn't know how to express their problem.*  *Most of the time, because they are expressing it in lay person's terms.*  *They are just equating or relating it to the closest experience of that thing.*  *And so, they do not read textbooks, just like diseases, do not read textbooks. So, the patient just says, I feel this way. So, the patient's information is almost always unreliable*’. *‘So, medicine will teach that if you say something we learn how to tease it out of you. So, if you said you had pain for example, …… I will try to tease it out* [of you] *by saying tell me more about this pain and I will force you to give the* [pain] *scale.’*  On information provided by patients: *‘there will be a variation and you know if someone tells the story, it probably turns out slightly differently by everyone in the room’.*  *‘Patients change their story quite a lot…they're not making things up that it is just the way it is ....*[so] *I will modify the treatment based on new information’.*  *‘ We must trust what the patient is telling us. But a lot of the time we will get people coming and saying that they just don't feel right, so then it is up to us to pick out what they mean by that. And* [it takes] *a lot of observation skills to figure out what is going on’.*  *‘I would like to know why a diagnosis was made’.*  *‘I do have reservations. I would have to see trial data to know how good that algorithm is …. There's a lot of nuance to this [diagnosing]’.*  *‘I would want to know* [to know if] *to do further investigations*  *to confirm or deny that’.*  It's not that *‘I don't trust what a patient is saying to me…*[it’s that] *they might not know or cannot remember’.*  *‘You don't just ignore what patients say’*  *‘You would need to know that* [DAISY] *has been properly tested’.*    *‘I would be sceptical of a DAISY report’ as ‘I will not be able*  *to ask some questions’.*    *On trust in DAISY: ‘I suppose an objective assessment, like a clinically validated assessment of whatever it is doing* [to see] *whether it is in line*  *with what a healthcare professional would end up doing’.* |
| SLEEC Reliance on non-verbal and other physical cues and intuition | *‘I think with a lot of healthcare there are certain things that you could miss if you just go through something like an algorithmic way of dealing with things. There is a lot of what is called ‘gut feeling’ involved, especially when someone might present to the robot to do observations and that is fine, but they are just numerical values. Sometimes you just look at the person and you can tell they are really sick’.*    All within the context of *‘ what the patient tells us’* and *‘the background of what I can see, what I can observe, what I can sense’.*  *‘You use intuition …you can see people* [who] *look ghastly. You know, they look a bit grey or just when they are particularly quiet, it is the quiet ones that are a bit more poorly and things like that really’.*  *‘There are times when we go, they don't look right and [you] can't put a finger on it. But I know something is wrong...* [I] *suppose* [it's] *the sign of years of experience’.*    *I have a feeling ... so that means that on the patient’s* [account/observations]*... everything looks OK, but I just do not like the look of that patient. You know, sometimes nothing was* [indicating] *that, I just don't like what I'm looking at. Even though all the numbers are fine. DAISY will not be able to say that. DAISY* [will not have that discretion] *and would just say the patient says they are fine. The numbers say they are fine. So here you go, they are fine’.*  Reliance on non-verbal cues: *‘You can tell from a patient’s body language when they come in… there are a lot of non-verbal cues.. the way their bodies interact* [to] *factor in’.*  *‘But a lot of the time we'll get people coming and saying that they just don't feel right, so then it's up to us to pick out what they mean by that and a lot of observation skills to figure out what's going on’.* |
| Practitioner-patient relationship  (*how does technology affect this relationship*) | *‘There is a human factor that is removed by using DAISY’* |
| SLEEC Empathy | My practice of ‘*empathy changes from patient to patient*, to ‘*adapt to the way I respond to a patient depending on the patient within a situation’*    *‘A patient just wants to,* [like] *any human, wants to feel appreciated. You know, you walk into the room* [and say] *Hello. How are you? And how are you today? …to let the person feel like you are listening to them... And then to be seen very promptly and not to be kept waiting, and if they are going to be kept waiting to be given information regularly’.*  *‘It is listening to the patient…to understand why they are [seeing you]’.*  It is about *‘listening* *to* [patients] *and trying to give them the best care possible’.* |
| Process | Flexibility in the triage process: *‘it starts from when the patient walks through your door and you need to keep reprioritising, re-triaging until the patient leaves the hospital’.*    Rare cases: There are instances that ‘*are unusual or rare you bring your experience in, but most of the time you just work at standard code level without really thinking’. ‘Some cases that are sort of rareish or they have strange treatments or for example, there is one presentation where we actually prescribe* [for example] *Coca-Cola’....It does not happen often but when it does happen I remember the last time this happened, we gave* *Coca-Cola and people around you say you are joking’.*    On problems in the A&E: *‘ I think the biggest problem is an overcrowded department’.*    *‘I know that we have been having quite a bit of a rough week recently, with very long waiting times, … the nurses would triage them* [by] *taking a very, very, very mundane and brief history, sometimes just a couple of words’.*    On wait times: ‘*Usually the patients just end up waiting in line to be seen by a doctor. It can range from give or take 2-3 hours up to 9-10 hours.’*    *‘I think* [DAISY] *can really try and streamline the process’.*    Good practice is about *‘communication, just keeping them up to date because they feel helpless,* [about telling them] *what's going on’.* |
| SLEEC Reassurance and managing expectations | *‘You know that the biggest intervention that we give our patient is reassurance. Somebody will attend to you* [and expects you] *will sort the problem out. That is the biggest weapon that we have ... .we say we are going to sort the problem, or we will try our best to solve the problem’.*  *‘Just comforting and reassuring the patient is important because sometimes we do not know the answer’.*  *‘I think we also have to be honest when we don't know the answer’.*  *‘Patients can say they feel absolutely ghastly and yet they have normal observations. So, although, I am sorry they feel rotten, their body is dealing with it really well, and don't need to come in, or need antibiotics’* |
| SLEEC Managing ‘difficult’ patients - importance of social skills | *‘Sometimes they need to feel like they have been seen or something is being done’.*    *‘If* [the patient] *is not comfortable, we can stop it and I will get a human or another clinician to see you’.* |
| SLEEC Explainability and transparency in the diagnosis and the report | ‘*It is important to be fully transparent* ’    *‘Don't think you should ever not tell them what you are looking for’*    *‘I think if she has to break bad news to patients it’s going to be difficult territory to navigate round’.*    On information patients want to be told: ‘*I think what the diagnosis is, any treatment they need, and probably the duration of symptoms’.*  Most patients want ‘*a diagnosis, which is not always the case’.* [not always possible]    *‘You need a plan, you have to say we are doing this, this and this. And if this, this and this does not work, then you go see your GP or you go see if it's a therapist or you. So, if you don't have an immediate diagnosis, you need to plan for what happens next’.* |
| SLEEC Privacy and disclosure of sensitive information | *‘....DAISY will go objectively and say I don't care what the patient looks like, this does not sound right. The other advantage that I'm guessing from a robotic thing we've discussed is some of the questions that we're supposed to ask….our patients, we don't ask them, but DAISY will…..And to be honest, you might feel more comfortable responding to a robot about something like domestic abuse or something embarrassing’.*    *‘If it is just a curtain between you and the next person, I think we have to be realistic that ..drawing a curtain round does not magically block all the sound’.*  *‘You know there are certain things people will not necessarily say at the front desk, but if it is in the privacy of the room they might say’.*  *‘One aspect that needs to be looked after would be the data safety and confidentiality. ..I assume that DAISY would be connected to some kind of cloud back end* [and] *that it needs to be ensured that it is quite secure and safe because if there is any kind of data breach, that would kind of lead to…game over…* [in the event of] *a data leak and all the patient’s data would be compromised’.*  *‘It's not private….it's stopped some shared information, …it's not as good as we would like it to be’.* |
| SLEEC Equality and implicit bias - assumptions as simply heuristics | *‘Even though medicine teaches that you must never, ever stereotype people, irrespective of their gender, race, religion, .. you are supposed to treat everybody the same. But we are human beings, so patients come to your door, and the first thing you do is stereotype them. But that stereotyping helps a lot, the only thing you now must do is to ensure that your stereotyping is masked. I am just guessing because if you do not stereotype you would not be able to get through the day. And sadly, so we must do it.’*    *‘Some presentations will trigger a few things. For example, if I saw a patient with burnt fingers. So, I sort of imagined that they have smoked all their lives and if they have, I am expecting them to have chest problems. And you know, if I see people that are malnourished and they look a bit higher or have the smell of alcohol, I think they can have liver problems. So, you will, you will have to stereotype. Otherwise, medicine would not work.’* |
| SLEEC Cultural and social sensitivities | *‘The way we treat people with our cultural sensitivity metre is very, very different’ .*  *‘We get lots of patients that do not speak the language that we speak. So, we struggled to communicate with them… if DAISY could* [communicate] *in* [various] *languages, even though they have interacted with the robots in whatever language, I get a report in English.’*  *‘Sometimes we have a language barrier, … English is not necessarily their first language, so we might have to explain things differently’.*  *‘Patients with hearing aids or who are a bit hard of hearing. DAISY may not necessarily be well tuned to something like that if* [such patients] *are not able to properly articulate what they want to say’.*  *‘We have different dialects and accents and some people have got a much stronger accent… it might be a bit more difficult for DAISY to interpret what they are trying to say. So, it might not work as well or as intended with specific demographics’.*  *‘I like to make accommodations for cultural sensitivities in my interactions with patients’.*  *‘For privacy we have curtains that we can pull around’.*  For example: *‘elderly people with dementia or someone with special needs nearly always come in with a carer. Although you try to ask the patient themselves all the questions, often it is the carers that have the knowledge, and know everything about the* [patient’s] *care’.* |
| Over- and/or underreporting symptoms | *‘I suppose someone who says they have got 10 out of 10 pain and then they are sat texting on their phone looking quite happy’.*    *‘.. there is probably some age demographic variation, … at times old people who probably do underreport more. I think it is… because of ..* [a] *stiff upper lip kind of thing. Some of it is stoical, and you know, we always have people, but if you see a farmer, that probably means there is something really badly wrong. Because they are a group of people who never come to see us, and if they do it is normally because they are probably not in a good place’.*    *‘When I do consultations,* [I try] *to holistically take in everything that the patient is coming with and use all of that to come up with a management plan for them’.* |
| Report and preliminary diagnosis | *‘If I get a report from DAISY I will look at the final diagnosis, but I think I will do what I normally do which is* [to establish] *how did you get to that diagnosis to see if I have a different opinion. If I have a different opinion I will say straight away, if I do not then I will …continue on that path [it] seems reasonable’.*    *On the question of having a different opinion to the DAISY report: ‘I would do what I would always do, which is get another human doctor to review and see if they can come up with a different diagnosis. Which is what happens in real life’.*  *‘I* [would like to know] *the drugs* [a patient] *is on’.*  On the report: *‘For me… to look at it, it has got to be short and succinct’.* |
| SLEEC Medical liability and duty of care | ‘*They have come to us for a reason, we need to see them all, so you cannot use it as a redirection tool’*. |
| Shortcomings in using DAISY | ‘*Medicine is an art as well as a science*’    Patients may ‘*say I could not tell my whole story or their story could potentially be misinterpreted*’    *‘I feel like you cannot really build a rapport with a robot’.*    On detecting subtleties in the information patients provide such as abuse, addiction etc: ‘I *think an automated system might have a downfall in those sorts of cases*’    *‘I don't think you would be able to trust everything at face value, at least not to begin with’*    Patients may be reluctant to change*, ‘especially older patients some of whom are still not happy using telemedicine’.*    In making a diagnosis: *‘ you use all your senses and then* [how can you] *design a system that does not have a sense. And the answer is very simple. As you know, as a junior doctor, I did not use my senses. DAISY can never be* [that]*.. I do not know, maybe somebody will design that.* [DAISY] *is not a consultant who uses their senses and smells the patient and feels if you know what I mean’.*    *‘The trial data must be location specific…. There may be cultural elements to it. If we introduced* [DAISY] *in a different country, we would get different evidence…*[for example] *health reported symptoms may be slightly different’.*    *‘.. in terms of cost, I think that might be one of the major things with incorporating DAISY into healthcare and in terms of whether its staff members need to be taught how to use it or navigate around it and the maintenance cost. So … the benefit of DAISY and what it brings into the A&E triaging system, needs to outweigh the cost of having it, purchasing it, and maintaining it in the long term. That would be like one of the key points to address before roll-out to most healthcare facilities’.*  *‘I don't think I would be a fan of the robot. I would want a person. This in my opinion’.*  *‘People* [who aren’t] *very computer literate …would never have a clue on how to use it’.* |
| Benefits to using DAISY | ‘*it could speed things up*’ and ‘*it might prevent people from sitting in the waiting room*’    *‘I think that* [DAISY] *would be beneficial. Patients would be seen by something quicker and would be able to triage and* [establish] *red flags and those* [patients] *who are really unwell, and then they could be alerted and put to the top of the list’.*    *‘I think* [DAISY] *would be quite beneficial. …I don't think DAISY in itself needs to be replacing anyone but* [I] *feel like it can do a lot in terms of streamlining what we do’ .*  *‘*[Although] *DAISY is not going to take a full, thorough history, but if it is able to take just the basics for me and I can then sort of just touch up on what I think is missing from that that would reduce the length of time that you spend with the patient and obviously if the investigations get ordered earlier, much earlier, then obviously I can try and tie up that entire consultation. So I've got all the evidence I need, all the investigations I would have ordered, I have the history with me, I have my clinical examination findings and I can .. combine all of that into what I think is going on and decide on what management the patient needs or whether they can go back home or whether they might need to be admitted’.* |

**Supplementary Material, Table 2 : Interviews - Practitioners Questions**

Part I: Status quo (what is done now, how are decisions made)

· From your perspective, can you describe the current (typical) A&E triage process after a patient has checked in with the receptionist

· Describe how you would (typically) review/diagnose the patient – when would you get involved?

*Schema (questions relating to experience and knowledge)*

- Are you aware of considering past experiences of previously similar events when interacting with patients?

- What expectations do you bring to the process (if you are aware of any)?

- FOR JUNIOR DRS:

o Is this situation comfortably within your experience (if not, why not)

o What (if any) training do you utilise (i.e., any specifics other than general medical training)

*World (questions relating to information obtained from the environment surrounding the activity)*

- What external information would you utilise (where would it come from, what would it tell you)

- What physical cues (e.g., sights, sounds, smells) would you utilise?

- Would you receive information from others (if yes, who and how would it be received)

- What artefacts would be available to you (e.g., written documentation, equipment etc.) and what would be most important to you?

- Would you ever be uncertain about the reliability or relevance of the information presented to you?

- Is there ever information unavailable to you that would be useful to have? (If so, what)

- What would influence/impact the trust you had of your patient?

- What information would you need to repair any lost trust in the patient?

*Action (questions relating to actions taken)*

- What are your decision options, what would influence these the most?

- What physical actions would you take?

- Would you communicate with anyone? (If so, who?)

- What inputs would you make into what technological systems?

- How would you evaluate and interpret the information available to you?

- Would you be following known conventions? (yes, what / no, why not?)

Social, Legal, Ethical, Empathetic, Cultural (SLEEC) considerations

- What cultural sensitivities and considerations do you accommodate for/make in your practice?

- How do you currently ensure you treat all patients equally and equitably?

- In your experience, what is the most important information that the patient wants to know/be told?

- How do you account for disabilities/mental incapacities (other outliers) in your practice?

- What does care, consideration and empathetic practice look like to you?

- How is privacy achieved in your current practice?

- What reliance do you place on non-verbal cues?

- Do you rely on intuition when assessing a patient?

- Do you find over- and under-reporting of symptoms? And if so, what alerts you to this?

Part II: Questions relating to the DAISY-generated Report

*Questions relating to Trust in the report*

- What would you use this report for?

- What would you be relying on the report for? (and not relying on the report for?)

- What information would you need to trust the report?

- How could trust in the report be strengthened?

- What would influence you/give you reason not to trust the diagnosis report?

- What could the most negative outcome be from an inaccurate report?

*Schema questions*

- FOR JUNIOR DRS:

o Is this situation (reviewing diagnostic reports) comfortably in your experience, or were there parts unfamiliar to you?

o Is there any specific training (other than general medical) that you are utilising?

- What (if any) expectations would you be bringing into this situation?

*World questions*

- What piece(s) of information would you place most importance on (why)?

- When would you be concerned about the reliability of the report?

*Action questions*

- What are your decision options? What would influence these the most?

- Would you communicate with anyone? (If so, who?)

- How would you evaluate and interpret the information available to you?

- Would you be following known conventions? (yes, what / no, why not?)

#### **Part II b) reviewing patient after reading DAISY report**

*Schema questions*

- FOR JUNIOR DR:

o Is this situation comfortably in your experience?

o What training (over and above general medical training) would you be utilising?

- What (if any) expectations would you be bringing into this situation?

*World questions*

- What would you be looking at/for (in the patient) when reviewing them with their report?

- What information would you place most/least trust in?

- What physical cues (e.g., sights, sounds, smells) would you utilise?

- When would you be concerned about the status of the patient?

- What physical actions would you take?

*Action questions*

- What are your decision options? What would influence these the most?

- What key features of the report and patient would you compare?

- What would you be relying on the report for? (and not relying on the report for)

- If there were discrepancies, who or what (patient, report, yourself) would you be most likely to trust and why?

- Would you communicate with anyone? (If so, who?)

- Would you be following known conventions? (yes, what / no, why not?)

**Supplementary Material,** **Table 3 : Social, legal, ethical, empathetic, and cultural considerations**

| **Social** | Apprehension around patient’s ability to use technology (particularly the elderly) | Inclusion and diversity of patients who have access to technology (computer literacy and ability to use technology) | Societal impact of bias in the algorithm/data |
| --- | --- | --- | --- |
| Legal | **Regulatory issues**  Safety and efficacy of the algorithm (testing, validation, approval, and confidence in the system)  Sufficiency of oversight mechanisms (algorithm quality/updates/location sensitivity, etc) | **Data Privacy/Protection**  Quality, accuracy, and credibility of input data  Lawful processing of sensitive personal data | **Legal liability**  Duty of care Responsibility and accountability if something is missed/misdiagnosed |
| Ethical | **Explainability** of how diagnosis is suggested/made and why (justifiable reasons) | **Equality and equity** considerations/neutrality | ‘**Virtues**’, such as patience, tolerance, compassion |
| Empathetic | Reliance on **non-verbal and other physical cues and intuition**, social skills | **Responsivity and adaptivity** of the system to individual cases | Ability to **probe further questioning** |
| Cultural | **Language** barriers | Cultural and customary **sensitivities and appropriateness** | Managing **expectations** |
